# Challenges in Organizing Trauma Care Systems in India

**DOI:** 10.4103/0970-0218.45383

**Published:** 2009-01

**Authors:** Amit Gupta, Ekta Gupta

**Affiliations:** Department of Surgery, JPN Apex Trauma Center, All India Institute of Medical Sciences, New Delhi, India. E-mail: amitg70@hotmail.com; 1 Department of Microbiology, Maulana Azad Medical College, New Delhi, India

Sir,

Injury and trauma, often used interchangeably, represent a major health problem worldwide. Everyday around the world almost 16,000 people die from various injuries. Injuries represent 12% of the global burden of disease.(1) Road traffic injuries are a major cause of mortality: 22.8% in the overall burden of death related to injuries.(2) It is startling to note that the lower and middle income group countries (which include India) contribute about 90% of the global burden of injury mortality, thus highlighting the disparities in outcome of trauma between the high, middle, and lower income nations. Injuries affect the productive youth of the country. In addition to excess mortality, there is a tremendous burden of disability from extremity, head, and spinal injuries in developing nations. The more tragic fact is that injury is the third most important cause of mortality and the main cause of death among 1 to 40-year-olds. Therefore, trauma effects the productive youth of the country, which is otherwise healthy and free from chronic disease. Road traffic injuries represent only a fraction of the trauma spectrum. In India, most of the available literature regarding trauma epidemiology is pertaining to road traffic injuries(3) and there are hardly any studies done on the other causes of trauma. Trauma is caused by a wide variety of risks e.g., fall (common in pediatric patients), agricultural-related injuries, firearm injuries, poisoning, burns, drowning, intentional self harm (suicides), assault, falling objects, natural- and man-made disasters.

The improved survival and functional outcome among injured patients in developed countries can be partly attributed to high-cost equipment and technology. Much of this high-end technology is unaffordable and unavailable to victims from developing nations. However, much improvement in the outcome of trauma patients has come from improvements in the organization of trauma care services in the form of developing trauma systems in given geographical areas. The improvement and organization of trauma services or trauma systems is a cost effective way of improving patient outcome and is achievable in almost all settings.(4,5) Proper organization of these systems reduces the time between injury and the definitive treatment thereby reducing morbidity and mortality. In India, such a trauma system is almost non-existent and even if present in some urban areas, lacks the cohesive effort required.(6)

The organization of a trauma system has four impact pillars: organization of pre-hospital care facilities, hospital networking, communication systems, and organization of in-hospital care (acute care and definitive care). An integrated approach is required at all levels: human resources (staffing and training), physical resources (infrastructure, equipment, and supplies) and the process (organization and administration). Compared to the western world, the trauma care services in India lack each of the elements listed above. Most of the physical resources for in-hospital care in terms of infrastructure and equipment are already available at secondary and tertiary care hospitals and need moderate upgrades. Therefore, the thrust areas in the field of trauma services are as follows:

1. Provide physical resources for pre-hospital care and communication systems.
2. Provide well-trained staff at all levels of care from pre-hospital to definitive trauma care. Providers should be well trained and should understand the critical needs of a trauma victim. Skill-based training programs for doctors as well as paramedical staff in Acute Life Support (ALS) procedures are needed.
3. Organize and integrate pre-hospital services with definitive care facilities (hospital) so that a patient is shifted to an appropriate facility in the shortest possible time.

The Government of India has planned this organization in an apex to the base format. The establishment of the Jai Prakash Narain Apex Trauma Center (JPNATC) at the All India Institute of Medical Sciences in New Delhi is a step forward in providing an apex institution for quality trauma patient care facilities, which will act as a role model for other institutions and centers providing trauma care in the country. More than providing the best patient care facilities, the role of this apex trauma center has been envisaged as an apex research and training institution that will help the nation's administrators formulate policies regarding the organization of trauma care facilities throughout the country.

It should be once again emphasized that the establishment of innumerable trauma centers with heavy financial burden should not be the goal of policy makers. Instead, upgrading existing hospital infrastructure to treat severely injured patients should be undertaken. Training of manpower in acute care and pre-hospital services should be a priority. Proper organization and administration of trauma services along with legislative backup will go a long way in strengthening India's essential trauma care services.
